# Hematological Malignancies and Arterial Thromboembolism

**DOI:** 10.1007/s12288-019-01085-x

**Published:** 2019-01-28

**Authors:** Nathan Visweshwar, Michael Jaglal, Lubomir Sokol, Benjamin Djulbegovic

**Affiliations:** 1 Division of Hematology, University of South Florida, Tampa, FL 33612 USA; 2 Division of Medical Oncology, Moffitt Cancer Center, Tampa, FL 35316 USA; 3 Division of Medical Oncology, City of Hope Cancer Center, Duarte, CA 91010 USA

**Keywords:** Thrombosis, Arterial, Hematological malignancies

## Abstract

Established guidelines exist for prevention and treatment of venous thromboembolism in hematological malignancies, but none for arterial thromboembolism. However, arterial and venous thromboembolism share the same provoking features—including altered procoagulant factors and defective fibrinolytic system. The morbidity for arterial thromboembolism is increasing in hematological malignancies, with the advent of immunomodulatory and targeted therapy. However, survival rate for hematological malignancy is improving. Consequently, as patients with hematological malignancies live longer, comorbidities including diabetes, hypertension and dyslipidemia, may accentuate arterial thrombosis. Thus far, the scientific literature on prophylaxis and treatment for arterial thromboembolism in hematological malignancies is limited. This review highlights the pathogenesis, incidence and clinical features of arterial thromboembolism in hematological malignancies.

## Introduction

In peer-reviewed literature, there is wealth of information available for venous thromboembolism (VTE) for hematological malignancies, but only limited data available for arterial thromboembolism (ATE). Mortality has decreased in hospitalized patients for VTE after enforcing thrombo-prophylaxis, but this has not affected the ATE events. Approximately 25% of the literature in thrombosis, appears to be in ATE [1]. The incidence of ATE is increasing with the advent of biological agents, targeted therapy with tyrosine kinase inhibitors and immunomodulatory agents in hematological malignancies [2, 3]. In hospitalized neutropenic patients from 1995 to 2002, there was 124% increase in arterial events when compared to 36% increase in venous events (*p* < 0.0001). The in-hospital mortality was greater in arterial [odds ratio (OR) 5.04] than venous thromboembolism [OR 2.01] [4]. In a retrospective analysis of patients receiving cisplatin-based chemotherapy for lymphoma, ATE was found in 8.3% of patients, and combined VTE and ATE was seen in 3.0% of patients [5]. Overall, there is a higher incidence of ATE in patients with previous history of deep vein thrombosis—with a relative risk for myocardial infarction of 1.6 and for stroke 2.1. In patients with pulmonary embolism, relative risk for myocardial infarction is increased by 2.6 and for stroke 2.9 [6, 7]. Most of the patients with hematological malignancies and ATE have no history of diffuse vascular disease accounting for thrombosis [8]. Patients with unprovoked VTE are at risk for ATE in hematological malignancies [9]. Accelerated coronary atherosclerosis, heart transplant atherosclerosis, vein graft disease, coronary restenosis after angioplasty may be the presenting manifestation of ATE in patients with hematological malignancy [10]. Apart from occlusion of major vessels, microvascular thrombosis may manifest as confusion, metabolic encephalopathy, widespread myocardial microthrombosis, thrombotic thrombocytopenic purpura or microemboli in skeletal muscles presenting as myositis in hematological malignancies [11]. The mechanism for ATE in hematological malignancies has not yet been characterized.

## Pathogenesis

**Fig. 1:**
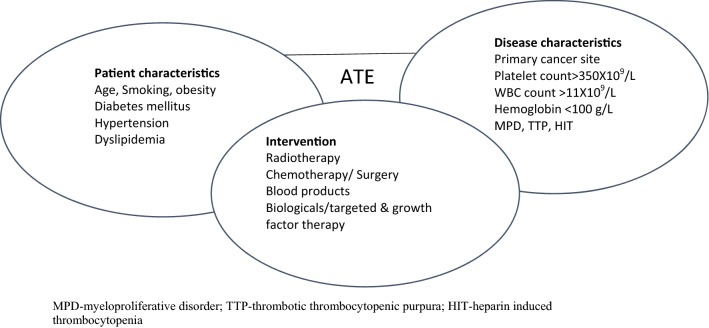
Interaction of patient characteristics, disease characteristics and intervention in ATE of hematological malignancies

The pathophysiology of ATE in hematological malignancies is not yet scientifically proven [12]. Several factors have been implicated in the prothrombotic state associated with ATE, including abnormal coagulation pathway, microparticles, cytokines, soluble P-selectin, elevation in coagulation factors, thrombocytosis and leukocytosis [13]. There appears to be better understanding of ATE in patients with myeloproliferative disorders, chronic myeloid leukemia (CML) and in myeloma. Nonetheless, most of the factors precipitating VTE, especially unprovoked VTE appears to trigger ATE—Fig. 1 [9, 14, 15].

### Abnormal Coagulation Pathway

**Fig. 2:**
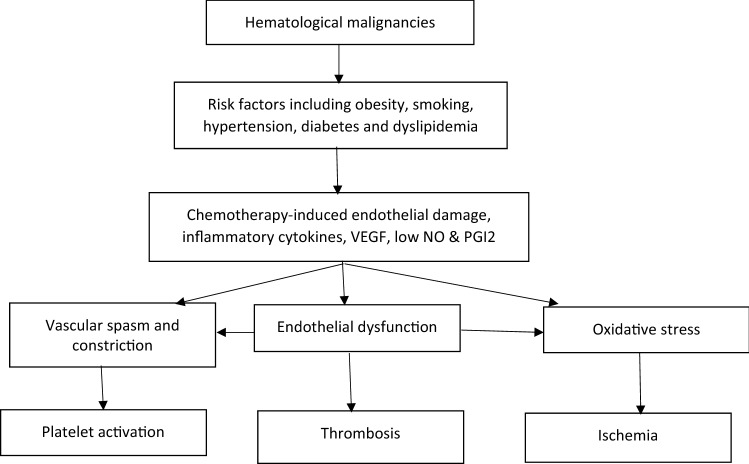
Interaction of endothelial dysfunction, risk factors and cytokines in hematological malignancies

The pathophysiology of ATE in hematological malignancy appears to be very similar to VTE, keeping in line with the three tenets of Virchow’s Triad, namely alterations in flow, abnormal cell and molecular properties and altered blood vessel walls (Table 1). The endothelial damage (Fig. 2) is exacerbated by tissue factor (TF) shed from microparticles of tumor tissue and activates platelets. This, in turn, activates the extrinsic pathway, binding factors VII and X, triggering thrombin generation [16, 17]. The activated platelets also release short-chain polyphosphates (SCP). The SCP derived from activated platelets acts as a cofactor for the inhibition of tissue pathway inhibitor alpha (TFPIα), suggesting that activated platelets in a hemostatic plug may contribute to the hemostatic role of FXI not only by enhancing the activation of FXI by thrombin and the activation of FV and FX by FXIa, but also by promoting thrombin generation through neutralization of TFPI [18]. In polycythemia Vera, increased adhesion of RBCs due to abnormal activation of adhesion proteins in RBCs to endothelium appears to contribute to thrombosis [19]. Photomicrography shows the red blood cells, leukocytes and platelets participate in development of ATE. Their mutual interaction appears to be important for thrombus formation [20]. The mechanisms of ATE in myeloma patients are multifactorial and include increased levels of procoagulant factors such as von Willebrand factor and factor VIII and high levels of inflammatory cytokines such as interleukin 6 and tumor necrosis factors [21, 22].

**Table 1:** Virchow’s Triad influenced by malignancy

Virchow’s Triad	Contributing factors
Altered flow properties	a. Viscosity (Myeloma/MPD)
Abnormal cellular and protein molecules	a. leukocytosis, beta thromboglobulin, P-selectin
	b. Prothrombotic state—microparticles, VWF, Fibrinogen, VIII
	c. Altered fibrinolysis—decreased tPA
Altered blood vessel wall	a. Endothelial damage—increased soluble E selectin, E-cadherin, laminin and beta thromboglobulin
	b. Vascular proliferation—galectins, VEGF

*MPD* myeloproliferative disorder, *tPA* tissue plasminogen activator, *VWF* von Willebrand factor, *VEGF* vascular endothelial growth factor

### The Role of Platelets and Polymorphonuclear Cells

Polymorphonuclear leukocytes play a major role in pathophysiology of ATE in myeloproliferative disorders. Polymorphonuclear leukocytes promote thrombosis through interactions of several adhesion molecules with other blood cell components. Their activation is associated with thrombotic events accentuated by Janus kinase (JAK2) mutational status [23]. Once activated, neutrophils release reactive molecules that can induce endothelial functional changes [24]. Polymorphonuclear neutrophils releasing extracellular DNA traps contributing to ATE is being recognized as a cause of cerebrovascular and cardiovascular dysfunction seen in arterial thrombosis of hematological malignancies [25]. Platelets appear to play a vital role in ATE of hematological malignancies. Once activated, platelets change shape and degranulate to release growth factors and bioactive lipids into the blood stream. This cyclic process recruits more platelets and increases thrombogenesis. The inflammation/coagulation from malignancy including hematological malignancies activates neutrophils and induces them to form neutrophil extracellular traps (NETs). The NETs either directly or through the consequences of platelet activation causes thrombosis. This may lead to widespread myocardial microthrombosis, presenting as ischemic episodes with elevated levels of plasma troponin [26].

### Abnormal Cellular Elements and Protein Molecules

The mechanisms for increased adhesion include overexpression of adhesion molecules such as selectins, which promote RBC adhesion to endothelium via laminin. In CML, tyrosine kinase inhibitors (TKI) including ponatinib inhibits numerous off targets, including vascular endothelial growth factor (VEGFR1-3), which may account for the systemic effect on the vasculature including arterial hypertension [27]. However, ponatinib acts as a platelet antagonist, and its effect on the vasculature is not secondary to platelet activation [28]. In myeloma there are high levels of inflammatory cytokines such as interleukin-6 and tumor necrosis factors [21, 22]. The JAK2 V617F mutation appears to increase proneness for thrombosis even without overt chronic myeloproliferative disorders [29, 30]. Platelet interaction with P-selectin demonstrates the integral role platelets play in the development of cancer-associated thrombosis [31].

## Hematological Malignancies and ATE

### Myeloproliferative Disorders

#### Essential Thrombocythemia, Myelofibrosis and Polycythemia Rubra Vera

In essential thrombocythemia (ET), ATE occurs more commonly than venous thrombosis [32]. The cumulative incidence of ATE in myeloproliferative disorders ranges from 54 to 80% [33]. The incidence of thrombosis at the time of diagnosis of patients with polycythemia rubra vera (PV) and ET is 9.7–38.6% with 64–96.7%, occurring in the arterial bed [34]. The overall cumulative rate of cardiac death was 7.2%, in myeloproliferative disorders [35]. It is postulated that JAK2 mutation leads to constitutive activation of the JAK2/STAT signaling pathway in myeloproliferative disorders, which in turn extends the inflammatory response or vice versa [36]. The risk of thrombosis in ET has been predicted by thrombosis history, older age, cardiovascular risk factors and JAK2V617F [37]. Extreme thrombocytosis (platelet count > 1000 × 10^9^/L) was predicted as a risk factor for thrombosis, but this was proven inaccurate by the International Working Group studies, which found a reduced risk of arterial thrombosis in patients with very high platelet count in ET [38]. This can be explained by the occurrence of acquired von Willebrand syndrome in ET patients with extreme thrombocytosis, consistent with previous reports [39, 40]. In patients with ET a positive correlation was observed between JAK-2 V617F mutation, that facilitates erythropoietin receptor signaling, and thrombotic events, although the mechanism involved is not clear. In patients with PV, arterial hypertension and age 50–60 was associated with 2-fold increase of arterial thrombosis [41]. In patients with myeloproliferative disorders, ATE was reported to be responsible for the ischemic stroke, myocardial infarction and peripheral arterial occlusion [42]. Abnormalities of blood cells, activation of neutrophils and platelets, and a hypercoagulability state, can all act in conjunction to lead to thrombosis in myeloproliferative disorders [43]. In patients with PV and essential thrombocythemia, cytoreduction protects against recurrent thrombosis [44].

#### Myelodysplastic Syndrome

In myelodysplastic syndrome (MDS), the burden of thrombotic events does not seem much higher than that of general population. This may be related to the low incidence of thrombosis due to high-frequency with thrombocytopenia and severe anemia. However, no clinical trials are available and retrospective studies are limited in size [45, 46]. The vascular risk seems to increase significantly when chemotherapeutic drugs such as thalidomide or lenalidomide are used with erythropoiesis- stimulating agents. But when erythropoiesis- stimulating agents are used alone in MDS, there does not appear to be an increased thrombotic risk [47]. Thrombosis related to MDS, has been associated with thrombotic thrombocytopenic purpura with occlusion of cardiovascular and abdominal vasculature [48]. Association of trisomy 8 in MDS has been reported as a risk factor for intestinal ulcers and thrombosis [49]. Acute Myocardial Infarction caused by thrombotic microangiopathy complicated by MDS needs antiplatelet or anticoagulant therapy, despite severe pancytopenia and increased bleeding risk [50].

#### Chronic Myeloid Leukemia

Patients with chronic myeloid leukemia (CML), if properly managed, have an expected life span like the rest of the general population [33]. Proper management of CML often involves treatment with one of the tyrosine kinase inhibitors (TKIs). Pulmonary hypertension has been reported in patients treated with dasatinib. Dasatinib-induced exudative pleural effusion, a frequent adverse event occurring in 15% to 35% of patients [51, 52]. Ponatinib-associated vascular events (stroke, coronary artery stenosis, limb ischemia and occlusion, and venous thrombosis) occurred in 29% of patients [12]. Bosutinib-treated patients reportedly developed arterial hypertension [53]. In a systematic review, Haguet et al. found that 4.78% of patients developed arterial occlusive events with the new generation TKIs compared with 0.96% with imatinib. Ponatinib (OR 3.26; 95% CI 1.12–9.50), nilotinib (OR 3.69; 95% CI 2.29–5.95) and dasatinib (OR 3.32; 95% CI 1.37–8.01) are all associated with a higher risk of arterial occlusive events when compared to imatinib [54]. Nilotinib, dasatinib and ponatinib—are associated with arterial thrombosis, but no such adverse effects have been reported for imatinib. This includes coronary artery disease, peripheral vascular disease and cerebrovascular disease [55]. Thus, for example, nilotinib produces peripheral arterial disease in about 10% of patients [56]. Patients who develop peripheral arterial disease develop this complication early in their disease, suggesting that pre-existing risk factors exist for development of peripheral arterial disease, preferentially involving lower extremities and small vessels as seen in patients with diabetes mellitus [57]. In a multivariable logistic regression analysis comparing nilotinib, imatinib and no TKI, the imatinib-only cohort had decreased incidence of peripheral arterial disease versus the no-TKI cohort (OD 0.062; 95% CI 0.005–0.544) [58]. The exact pathophysiology of TKIs causing arterial occlusive phenomenon is not known, but direct pro-atherogenic and anti-angiogenic effects on vascular endothelial cells has been postulated [59]. Nevertheless, according to the population-based studies, the life expectancy of CML is now associated the normal life expectancy for most patients, provided that patients take lifelong TKI/anti-thrombotic treatment regimen [60].

### Acute Leukemia

Thrombotic complications in patients with acute leukemia occur frequently, significantly affecting morbidity and mortality [61]. Acute arterial occlusion in acute leukemia included cerebrovascular, cardiovascular and peripheral vascular occlusive disorders [62]. In 379 adult patients with newly diagnosed acute leukemia (ALL in 69 patients, acute promyelocytic leukemia (M3) in 31, and non-M3 AML in 279) 5 arterial thrombotic events were reported(AML type 5—in 2patients, AML type 3—in two patients, AML type 1—in 1 patient and none in ALL) [63]. In acute promyelocytic leukemia (APL), thrombosis as a presenting symptom at diagnosis (which responds to effective therapy) occurs in 9.6% and in other types of acute myeloblastic leukemia in 3.2% of patients. The following factors are related to a higher incidence of thrombosis in APL: leukocytes > 10 × 10^9^/L (9% vs. 4%, *p* < 0.01), M3-variant subtype (11% vs. 4%, *p* = 0.02), fibrinogen < 170 mg/dl (7% vs. 3%, *p* = 0.02) and hemoglobin > 10 g/dl (8% vs. 4%, *p* = 0.03). No significant relation was observed with CD2 or other surface antigens, as well as FLT3 mutations [64]. Rashida et al., found 94 cases of ATE in patients with APL, out of which 80% occurred at the time of diagnosis. The coagulopathy in APL is multifactorial, with both disseminated intravascular coagulation and primary hyper-fibrinolysis mediated largely by the malignant leukocytes [65]. The specific anti-leukemia therapy including use of all trans-retinoic acid has been shown to reverse coagulopathy on APL [42, 66]. In patients with acute lymphoblastic leukemia, at the time of diagnosis, the incidence was reportedly low—just 1% [67]. The mechanism of arterial occlusion in patients with acute leukemia apart from APL is not known. Suggested mechanisms for ATE in acute leukemia include the combination of hyperleukocytosis and aggregation of platelets leading to arterial occlusion [68, 69].

### Lymphoproliferative Disorders

#### Hodgkin’s Lymphoma

In Hodgkin’s lymphoma, accelerated coronary artery disease with increased mortality occurs with radiation to the mediastinum [70, 71]. Using the Surveillance Epidemiology and End Results (SEER) database from 2002 to 2011, the 6-month cumulative incidence of arterial thromboembolism in non-Hodgkin lymphoma was 5.4% (95% CI 5.1–5.8%) compared with 2.2% (95% CI 2.0–2.4%) in control patients (*p* < 0.001) [72]. Follow-up of a cohort of 7033 Hodgkin disease patients who were treated in UK—deaths from myocardial infarction was statistically significant (standardized mortality ratio [SMR] 2.5, 95% confidence interval [CI] 2.1–2.9), with an absolute excess risk of 125.8 per 100,000 person-years [71]. Analysis of the treatment and follow-up records of 377 Hodgkin’s disease patients followed from January 1964 to September 1972, who received mantle irradiation, but no planned chemotherapy reveals an overall supradiaphragmatic relapse rate of 21% with no vascular complications. Other complications of treatment included symptomatic pulmonary radiation reaction (20%), pericarditis (13%), Lhermitte’s sign (15%), and thyroid dysfunction (13%) [73].

#### Non-Hodgkin’s Lymphoma

In a retrospective review of acquired thrombophilia in lymphoproliferative disorders, Lechner et al. [74] analyzed 66 cases of immune-mediated thrombophilia in patients with lymphoma reported in the literature. They found 61 patients had lupus anticoagulant, out of which there were 7 patients had ATE. About 6.5% had a catastrophic antiphospholipid antibody syndrome. In their analysis, incidence of arterial thromboembolism was half of venous thromboembolic episodes. T cell lymphoma presenting as hyper-eosinophilic syndrome was associated with arterial thrombosis in 40%, with mixed arterial and venous thrombosis in 27% [75]. Patients treated with doxorubicin for diffuse large B-cell lymphoma develop not only cardiomyopathy, but also accelerated coronary artery disease with high cardiovascular mortality as a late complication with this agent [76]. Thrombotic occlusions of the popliteal and tibial arteries are reported in patients with Castleman’s disease [77]. Intravascular large B-cell lymphoma is associated with arterial occlusion [75]. Treatment induced microvascular induced neurotoxicity of chemo-radiation therapy of CNS lymphoma include: vincristine neuropathy, ifosfamide or cytarabine encephalopathy, radiation myelopathy, or radiation related cognitive impairment [78]. The risk of radiation induced vasculopathy or myelopathy following conventionally fractionated radiotherapy to the spinal cord for spinal cord involvement by Non-hodgkin’s lymphoma is extremely small [79].

### Bone Marrow Transplantation

Following hematopoietic stem cell transplantation, thrombotic microangiopathy was seen in 20% of kidneys at autopsy and an additional 15% had evidence of ATE [80]. Thrombotic microangiopathy and microthrombosis secondary to polymorphonuclear neutrophils releasing extracellular DNA traps contribute to organ dysfunction similar to major ATE. Renal failure occurs from thrombotic microangiopathy from calcineurin-inhibitors and total-body radiation [81, 82]. This manifests as rising serum creatinine, hypertension, progressive anemia, elevation of lactic dehydrogenase, low serum haptoglobin, thrombocytopenia and blood film showing schistocytes. Renal biopsy reveals glomerular podocyte injury, damage to endothelial cells along with swollen glomerular epithelium with fibrin deposition [83]. Apart from renal failure, patients may also have neurological and GI symptoms. Bloody diarrhea in thrombotic microangiopathy (from calcineurin-inhibitor therapy) may be difficult to clinically distinguish from acute graft versus host disease (GVHD). This may be of relevance, as patients in whom intestinal pathology demonstrated thrombotic microangiopathy may need to diminish, immunosuppressive treatment, since calcineurin-inhibitor therapy (rather than GVHD) may be contributing to the bloody diarrhea [84]. Plasma exchange, which is a potentially curative therapy in thrombotic thrombocytopenic purpura, has no proven efficacy in transplant related thrombotic microangiopathy. Post-transplantation microangiopathy treated with plasma exchange, response rates are generally less than 50%, and mortality rates among patients treated with this modality remain greater than 80% [85]. Blocking the complement system with eculizumab is currently the most effective treatment to circumvent the poor outcome in patients with severe transplant related thrombotic microangiopathy [86]. The mortality rates in patients who develop severe transplant related thrombotic microangiopathy are in excess of 80% [86].

### Plasma Cell Dyscrasias

#### Myeloma

Arterial thrombosis (coronary artery disease, cerebrovascular disease, myocardial infarction) occur during or soon after induction chemotherapy/immunotherapy for multiple myeloma (MM) [87]. The cumulative incidence of cerebrovascular thrombosis in MM is about 7.45% in 5 years [88]. In a prospective cohort study, Libourel et al. reported 5–12.5% of ATE in MM patients treated with Thalidomide. In this study, hypertension, smoking and elevated factor VIII levels contributed to the risk of arterial thrombosis [87]. The incidence of ATE was nearly 2-fold during first year of therapy for patients with myeloma [89]. Bowcock et al. reported 2 out of 23 patients on thalidomide for myeloma developed cerebral arterial ischemia [90]. Patients developed ATE during warfarin treatment, suggesting that warfarin is not sufficient as prophylactic treatment in MM patients who are at high risk for arterial thrombosis [87]. Prophylactic aspirin is used in myeloma to prevent both arterial and venous thromboembolic episodes [91]. The incidences of myocardial infarction and cerebrovascular events were 1.98% and 3.4%, respectively, in patients treated with lenalidomide and dexamethasone compared with 0.57% and 1.7%, in patients treated with dexamethasone alone [3]. The incidence of ATE with the newer agent, pomalidomide is not known. Bortezomib has a protective effect on thromboembolic phenomena in patients with myeloma, as platelet aggregation induced by the agonists were decreased after exposure to bortezomib. Patients may not routinely need thromboprophylaxis for combination therapy regimens with bortezomib in multiple myeloma [92]. However, with novel agents, the overall survival for patients diagnosed with myeloma between 2006 and 2010 has increased to 6.1 years [93].

#### Systemic Amyloidosis

Arterial thrombosis in AL-amyloidosis, especially coronary thrombosis occurs in nearly 50% of patients with 26% mortality [94]. Intracardiac thrombosis and thromboembolic events occurred in 26–33% of patients with primary amyloidosis (AL type) with preserved left ventricular ejection fraction [94]. AL-amyloidosis can occur de novo, or from excess light chain deposition from hematological diseases including multiple myeloma, Waldenström’s macroglobulinemia and non-Hodgkin’s lymphoma [95]. Mechanisms of ATE in cardiac amyloidosis include hypercoagulability, endothelial dysfunction, endomyocardial damage, direct myocardiotoxic effects and left ventricular diastolic dysfunction [96]. Antithrombotic treatment in AL amyloidosis is complicated by bleeding tendencies secondary to acquired factor X deficiency [97]. The treatment of ATE is directed against underlying disease process. This includes alkylator-based chemotherapy, immunomodulatory agents, proteasome inhibitors and stem cell transplantation after high-dose chemotherapy [98]. The emerging treatment in horizon for AL-amyloidosis includes a chimeric antibody reactive with many AL fibrils [99]. Other novel agents include small interfering RNAs is being explored as a treatment option in reducing the expression of the amyloid precursor protein. The in vitro studies show inhibition of synthesis of light chains in transfected cells, and in vivo there is reduction in the production of circulating free light chains [100]. In light chain amyloidosis, the most common type of amyloidosis treatment with bortezomib, lenalidomide, dexamethasone, renal transplantation followed by autologous stem cell transplantation, the 1- and 5-year overall survival has increased to 84% and 76%, respectively [98].

### Miscellaneous

#### Radiation Therapy

Ionizing radiation leads to release of superoxide, hydrogen peroxide and hydroxyl radicals which cause endothelial damage and activation of coagulation cascade [100]. Ionizing radiation leads to perivascular edema at 4–8 weeks and initiation of fibrosis after 20 weeks [101]. There was a 3.6-fold increased incidence of myocardial infarction at 19-year follow-up in patients with Hodgkin disease [102]. The release of cytokines including transforming growth factor beta, induces proliferation of fibroblasts and accelerated atherosclerosis [103]. The ‘oxygen effect’, is the radiation effect of the tissues secondary to superoxide radicles (less sensitive at lower level of oxygen). The dose, the technique (intensity modulated radiation therapy versus three dimensional configuration), extent of the vasculature exposed decides the extent of atherosclerosis and arterial thrombosis [103]. Radiation induced arterial thrombosis is secondary to accelerated atherosclerosis, leading to vascular events like stroke, coronary artery disease, and peripheral artery disease. This is dependent on the radiation dose and technique and extent of vasculature exposed [104]. There is a greater risk of radiotherapy-induced vascular diseases in younger patients when irradiating the mediastinum, in the treatment of Hodgkin’s disease, when compared to the elderly [105]. The incidence of coronary artery disease risk is proportional to the radiation dose to the heart and can be seen within 5 years of radiotherapy but typically presents in the second to third decade post-therapy [106]. The manifestation of atherosclerotic process in these vessels include: stenosis, thrombosis, and aneurysmal dilatation [107]. Occlusive arterial disease within the irradiated field with relative sparing of non-irradiated arteries is highly suggestive of radiation-induced occlusive arterial disease [104]. Variety of endovascular and surgical procedures are used to treat radiation-induced occlusive disease [108]. Hodgkin’s disease survivors are at 2- to 12-fold increased risk of CV mortality when compared to controls, largely attributable to myocardial infarction [71, 109]. Total body radiation used as conditioning regimen in bone marrow transplantation for myelodysplastic syndrome and acute leukemia (in particular acute leukemia in childhood), was not associated with ATE, especially coronary artery disease unless there was mediastinal irradiation [110].

#### Antiphospholipid Syndrome, Disseminated Intravascular Coagulation and Heparin-Induced Thrombocytopenia

In disseminated intravascular coagulation (DIC), ATE occurs in about 3% of patients with hematological malignancies. Without effective control of the underlying cause, treatment of DIC is not usually successful [111]. A higher rate of ATE was observed in patients with heparin-induced thrombocytopenia (HIT) and malignancy, when compared to patients with no underlying malignancy (odds ratio 13.6, 95% confidence interval 2.9–63.8) [112]. The antiphospholipid syndrome has been associated with hematological malignancies [113]. Lupus anticoagulant was present in 41% of patients with aggressive non-Hodgkin’s lymphoma which correlated with shortened survival [114]. Cerebral manifestations were most common and consisted mainly of cerebral infarcts and encephalopathy [114]. Platelet-leukocyte-endothelial aggregates generate HIT-specific thrombosis. Arterial thromboembolic episodes including stroke, acute myocardial infarction, mesenteric, renal, aortic and limb arterial occlusion can occur in patients with HIT [115].

#### Therapy-Related Arterial Thrombosis

Potentially fatal complication affecting mostly the kidneys and the brain microvascular changes occur with cisplatin, bleomycin and gemcitabine. Cisplatin induced endothelial vascular damage leads to coronary artery disease, but if this is dose dependent is not known [116, 117]. Vinca alkaloids and mitomycin C are associated with disseminated intravascular coagulation [118, 119]. Rituximab generally is a well-tolerated medication, but rituximab-induced coagulopathy with thrombocytopenia and disseminated intravascular coagulation is reported following rituximab administration [120]. Therapy with CD19-targeted chimeric antigen receptor-modified T (CAR-T) cells can be complicated by neurologic adverse events in patients with refractory B-cell malignancies. Patients with severe neurotoxicity demonstrated evidence of endothelial activation, including disseminated intravascular coagulation, capillary leak, and increased blood–brain barrier permeability [121].

#### Growth Factors and Arterial Events

The hematopoietic growth factors such as erythropoietin, granulocyte colony-stimulating factor and macrophage-granulocyte colony-stimulating factor have been implicated in ATE [117]. The erythropoietin receptor is widely distributed in endothelial cells, smooth muscle cells and cardiomyocytes. An increase in cardiovascular events, vascular access thrombosis, stroke and myocardial infarction, has been associated with erythropoietin [122]. Granulocyte colony-stimulating factor can cause acute arterial occlusion due to platelet aggregation [123]. Macrophage-granulocyte colony-stimulating factor (GM-CSF) causing thrombosis of common, internal and external iliac arteries has been reported in patients receiving chemotherapy [124]. Growth factors do not directly modulate endothelial cell function, but this may be related to activation of coagulation factors and hemostasis [125]. Macrophage-granulocyte colony-stimulating factor leads to the release of secondary cytokines, including tumor necrosis factor (TNF) and interleukin- 1 (IL-l), which are associated with alterations in coagulation [126]. Hematopoietic growth factors along with chemotherapy enhances endothelial cell reactivity to platelets causing ATE [127].

## Prediction and Risk Assessment Model for Arterial Thrombosis in Hematological Malignancies

There is no risk assessment model or predictive score available for predicting arterial thromboembolism in cancer patients [128]. The existing risk assessment models including Khorana, Vienna, Protecht, and Concho scores in venous thrombosis, are not applicable in arterial thrombosis [128].

## Algorithm for management of arterial thrombosis in hematological malignancies: Fig. 3

There are no standard guidelines for management of arterial thrombosis in hematological malignancies. Treatment of ATE is individualized, based on clinical condition of the patient, rapidity of the onset and the associated comorbidities. The underlying incriminating agent (TKIs, immunomodulatory agents, growth factors including erythropoietin), must be withheld and appropriate measures taken to improve the blood supply to the vascular territory. Treatment usually involves a multidisciplinary approach involving internists, intensivists, interventional radiologists and the surgical teams. The treatment algorithm, is based on the clinical scenario, underlying hematological disorder, pre-existing comorbidities, identifiable hypercoagulable state and previous treatment received by the patient. In the absence of clear data from randomized studies and guidelines for recommendations, our aim is to provide a rational approach to the management of ATE in hematological malignancies based on clinical experience, acknowledging that there is no evidence-based algorithm available for this entity.

**Fig. 3:**
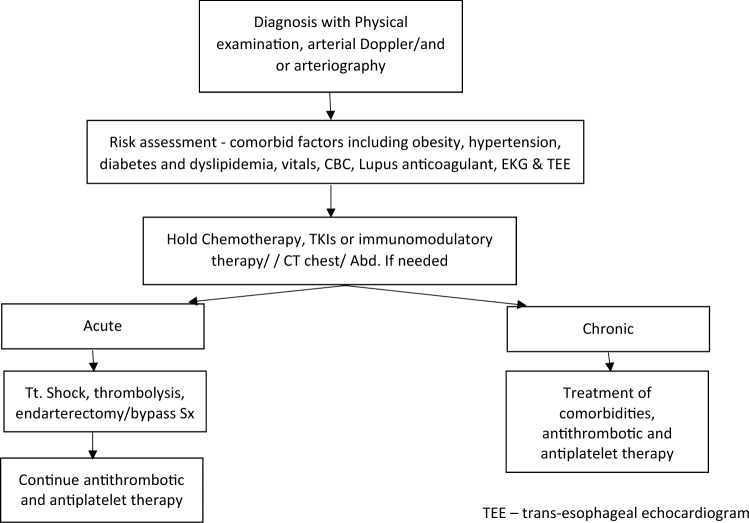
Proposed algorithm for management of ATE in hematological malignancies

### Prophylaxis

Primary prophylaxis for arterial thrombosis in outpatient setting of ambulatory patients is precluded by lack of data of the benefit, except in patients with chronic myeloid leukemia, myelofibrosis and myeloma. Primary prevention of arterial thrombosis with antiplatelet therapy is warranted for patients with myeloproliferative disorders, for patients receiving TKIs in chronic myeloid leukemia, immunomodulatory therapy for myeloma, age above 60, or with history of previous history of ATE. The role of anticoagulant therapy preventing ATE in hematological malignancies is not yet proven. Prophylaxis may be considered for patients receiving chemotherapy with previous history of DVT/PE, as studies have shown that previous history of venous thrombosis especially in unprovoked setting, leads to arterial thrombosis [129]. There appears to be emerging evidence for primary thromboprophylaxis, based on risk assessment with biomarker screening including P-selectin, CRP, factor VIII, prothrombin F 1 + 2, and TF-bearing microparticle levels in patients with cancer, but these are not yet validated [130].

### Management

There is no randomized controlled trial available to guide the clinician for the management of ATE in hematological malignancies. Treatment for ATE may need to be individualized (smoking cessation, controlling weight and treatment of co-morbidities). Occasionally, patients may have vasculitis or patent foramen ovale causing ATE, independent of hematological malignancies. After initial assessment, the medical management for acute arterial or venous thromboembolism appear to be similar—Fig. 3. The initial treatment is intravenous heparin or low-molecular-weight heparin [131]. Concurrent antiplatelet agents and/or statins along with anticoagulant therapy is suggested for ATE. Life-threatening arterial thrombotic events may need endoluminal revascularization and surgical procedures including angioplasty, thrombo-embolectomy and arterial bypass have mixed results [6].

In non-life-threatening ATE, the treatment paradigm includes thrombolytic therapy with streptokinase or urokinase or combination of both, LMWH and newer agents including recombinant tissue factor pathway inhibitor and anti-tissue factor monoclonal antibodies [132, 133]. Promising agents under evaluation for micro-thrombosis secondary to thrombotic thrombocytopenic purpura include, caplacizumab (an inhibitor of the glycoprotein-Ib/IX-Von-Willebrand factor axis), *N*-acetyl cysteine, recombinant ADAMTS13, and anti-plasmocyte compounds [134]. When long-term anticoagulation is advised, careful consideration should be given to the risk associated with therapy [131]. A conservative nonsurgical therapeutic approach was suggested for patients with peripheral arterial occlusion, because of poor outcome with increased post-operative mortality (as high as 80–100%) [8].

### Prognosis

Arterial thrombosis is associated with higher mortality when compared with age adjusted controls with systemic vascular disease in malignant hematological disorders [132]. Even with specific surgical intervention of arterial thrombosis in active malignancy, the outcome is very bleak [8]. About 80% of patients die within 1 year, compared to 80% survival during the same time period without cancer from atherosclerotic vascular disease [8, 135]. In malignancy, including hematological malignancies, the survival rate from the time of presentation of arterial thrombosis was 50% at 3 months and 17% at 1 year [8]. Therefore, aggressive management for pre-existing risk factors including tobacco use, hypertension, dyslipidemia, increasing age, obesity, metabolic syndrome, renal failure, hyper-homocystinemia and diabetes mellitus is needed for patients with cancer, but value of this in preventing arterial thrombosis is questionable.

## Future Perspectives

The data on ATE in hematological malignancies is just emerging. From a preclinical stand point, the more we learn about the biology of ATE, the better we understand the mechanisms altered in hematological malignancies. This will also help us understand the role of targeted therapies incriminated in ATE. There are more novel therapies in pipeline, aimed to improve the overall survival of patients with hematological malignancies. We need randomized controlled trials for prophylaxis and treatment of ATE. There is a need for epidemiological studies to ascertain the incidence of ATE in malignant hematological disorders. This may help us with the appropriate prophylaxis and treatment of ATE in individual clinical entities.

## Conclusion

Arterial thromboembolism is a serious complication with high mortality in patients with hematological malignancies. The landscape appears to be bright for hematological malignancies with major breakthroughs in the treatment, including the next generation tyrosine kinase inhibitors, immunomodulatory agents, proteasome inhibitors and monoclonal antibodies. We need innovative approaches to decrease the incidence of ATE including: (a). appropriate prophylaxis (aspirin with ponatinib/immunomodulatory therapy) (b). identifying chemotherapeutic agents with increased incidence of ATE (cisplatin) (c). Concurrent use of agents (proteasome inhibitors and immunomodulatory agents) (d). avoiding precipitating agents (use of tobacco, habituating agents including cocaine) and (e). appropriate management of comorbidities including diabetes mellitus, dyslipidemia, atrial fibrillation and obesity. Some of these strategies have already proven to be successful. Early recognition and appropriate intervention may improve recovery and decrease mortality of ATE in hematological malignancies.
